# Seasonal variation of net ecosystem carbon exchange and gross primary production over a Loess Plateau semi-arid grassland of northwest China

**DOI:** 10.1038/s41598-024-52559-6

**Published:** 2024-02-05

**Authors:** Xueteng Zhang, Jianrong Bi, Di Zhu, Zhaozhao Meng

**Affiliations:** 1 Key Laboratory for Semi-Arid Climate Change of the Ministry of Education, College of Atmospheric Sciences, Lanzhou University, Lanzhou, 730000 China; 2 Collaborative Innovation Center for Western Ecological Safety, Lanzhou University, Lanzhou, 730000 China; 3 Field Scientific Observation and Research Station of Semi-Arid Climate and Environment of Gansu Province, Lanzhou, 730000 China

**Keywords:** Ecology, Grassland ecology

## Abstract

Grassland ecosystems store approximately one-third of the global terrestrial carbon stocks, which play a crucial role in regulating the carbon cycle on regional and global scales, but the current scientific understanding of the variation in net carbon dioxide exchange (NEE) on grassland ecosystems is still limited. Based on the eddy covariance technique, this study investigated the seasonal variation of ecosystem respiration (Reco) and gross primary production (GPP) from 2018 to 2020 in a semi-arid grassland on the Loess Plateau in northwest China. The results indicated that the annual cumulative average NEE value was − 0.778 kg C/m^2^, the growing season cumulative value accounted for approximately 83.81%, which suggested that the semiarid grassland showed a notable soil carbon sink. The correlation analysis revealed that soil temperature (T_s_) (R_Reco_ = 0.71, R_GPP_ = 0.61) and soil water content (SWC) (R_Reco_ = 0.47, R_GPP_ = 0.44) were the two main driving factors in modulating the variation of daily average GPP and Reco (P < 0.01). Therefore, the monthly average of GPP and Reco increased with the increase in T_s_ (R_GPP_ = 0.716, P < 0.01; R_Reco_ = 0.586, P < 0.05), resulting in an increase in the carbon sequestration capacity of the grass ecosystem. This study also showed that soil moisture has a promoting effect on the response of Reco and GPP to T_s_, and the correlation among GPP, Reco, and Ts was much stronger under wet conditions. For instance, the coefficient of determination of Reco and GPP with Ts under wet conditions in 2018 increased by 0.248 and 0.286, respectively, compared to those under droughty conditions. Additionally, the temperature sensitivity of Reco (Q_10_) increased by 46.13% compared to dry conditions. In addition, carbon exchange models should consider the synergistic effect of T_s_ and SWC as one of the main driving factors for theoretical interpretation or modeling. Under the potential scenario of future global warming and the frequent extreme weather events, our findings have important implications for predicting future CO_2_ exchange and establishing an optimal ecological model of carbon flux exchange.

## Introduction

Grassland ecosystems cover approximately 40.5% of the Earth’s land surface excluding Greenland and Antarctica and store about one-third of the global terrestrial carbon stocks. These ecosystems play a crucial role in regulating the carbon budget balance and carbon cycle processes on both regional and global scales^1^. Grassland areas experience cold winters and warm summers, characterized by low precipitation and high evaporation rates. According to various hydrothermal conditions, grasslands can be classified into four types: desertification grasslands, meadow grasslands, shrubland grasslands, and typical grasslands^2^. Grassland ecosystems around the world are primarily located in arid and semiarid regions, making them highly susceptible to global climate change^3^. These ecosystems have a short growth cycle, a rapid renewal rate, and relatively fragile productive capacity^4^. Ahlström et al. combined an ensemble of ecosystem and land-surface models with an empirical observation-based gross primary production (GPP) product, and demonstrated that the carbon sink of global terrestrial ecosystem was mainly dominated by tropical forests^5^. Whereas the trend and interannual variability of the sink are dominated by semi-arid ecosystems whose carbon balance was strongly linkage with general circulation-driven variations in both precipitation and temperature. Therefore, a comprehensive understanding of the seasonal variation patterns and mechanisms of carbon flux exchange characteristics is crucial for accurately assessing the regional carbon budget balance in different grassland types in semi-arid regions.

Net ecosystem carbon exchange (NEE) mainly refers to the changes in carbon exchange between terrestrial ecosystems and the atmosphere. It is influenced by factors such as plant photosynthesis, carbon storage in the canopy air, and carbon emissions from biological and abiotic respiration consumption in the ecosystem. It is determined by ecosystem respiration (Reco) and GPP^6,7^. Reco includes plant autotrophic respiration and soil microbial decomposition of soil organic matter, as well as litter respiration flux^8^. GPP refers to the quantity of organic matter produced by the fixation of carbon dioxide during photosynthesis in green plants, measured per unit of time and area^9^. Many studies have shown that various environmental factors, including both biological and abiotic factors, can affect the exchange of carbon flux in grasslands^10–12^. These environmental factors include air temperature, soil temperature (Ts), precipitation (Pre), soil water content (SWC), vapor pressure deficit (VPD), and surface cover characteristics. The different environmental factors that control carbon flux exchange have different influences on different time scale. Niu et al. studied the carbon fluxes of a desertification grassland in Inner Mongolia, China. They examined the responses of these fluxes to environmental factors on different time scales using random forest models and correlation analysis. The study found that photosynthetic photon flux density and soil temperature at a depth of 50 cm were important environmental factors in controlling the daily variation of NEE and GPP in most integration periods, whereas Ts and SWC were more important for Reco^13^. In meadow grasslands, photosynthetic active radiation is a dominant factor that affects the daily variation of NEE. However, in typical grasslands, shallow soil water content (at 5 cm depth) also plays a significant role in the daily variation of NEE^14^. Jia et al. investigated the seasonal and interannual fluctuations in Reco and its correlation with temperature, soil moisture, and GPP in a temperate semi-arid shrubland located in northern China. They believed that low soil moisture had little effect on Reco when Ta was below 15 °C, but it led to smaller Reco rates when Ta was above 15 °C^15^. The temperature range (or other environmental conditions) should be considered when examining the main and interactive effects of moisture and temperature on respiration. Leaf area index (LAI) was significantly positively correlated with Reco and GPP, whereas NEE was significantly negatively correlated with LAI. Shi suggested that the sink/pool relationship of the present ecosystem is largely influenced by rainfall, including its intensity and seasonal distribution^16^. For typical semi-arid grasslands, there have been many previous studies. For example, Yao et al. investigated the variations of NEE and the mechanism of environmental response on different time scales over the semiarid Loess Plateau in northwest China. They discovered that NEE was primarily influenced by soil moisture during the growing season, while soil temperature affected the changes in NEE during the dormant seasons^17^. Du et al. analyzed the carbon exchange characteristics and main environmental impact factors of grassland ecosystems in different locations over semi-arid regions. They indicated that the respiration of the ecosystems in semi-arid regions was primarily influenced by soil temperature and soil moisture. The start time, intensity, and temporal distribution of effective precipitation during the growing season jointly determine the function and the duration of net carbon absorption in semi-arid grassland ecosystem^18^. Some studies have also indicated that in certain semi-arid grasslands or under specific environmental conditions, there was no significant correlation between daily changes in carbon flux and photosynthetic active radiation or soil temperature^19,20^. These differences are due to the vast territory of China and the diverse natural and climatic conditions in different regions. Although previous studies have provided a foundation for studying the driving factors of carbon exchange in various grassland ecosystems, there is still a lack of understanding regarding the seasonal variation and dominant factors of carbon fluxes in the semi-arid typical grassland of the Loess Plateau in northwest China.

Soil temperature and moisture are two key factors that regulate plant distribution and productivity^21^. It is generally believed that global warming will accelerate photosynthesis and respiration^22^, promoting plant productivity^23^. Fu indicated that photosynthesis plays a crucial role in regulating ecosystem respiration on different time scales^24^. Additionally, temperature affects plant growth and distribution through physiological processes like photosynthesis and respiration^25–27^. Appropriate moisture conditions are crucial for promoting carbon flux, dry soil may limit Reco by reducing the activity of plants and soil microorganisms, as well as by limiting the diffusion of enzymes and carbon substrates in the soil^28,29^. High SWC may also limit Reco by reducing the soil oxygen concentration and aerobic respiration rate of soil organisms^30^. The interaction between water and temperature also results in changes in the carbon flux response process^22^. Soil moisture not only affects the scale of the ecosystem or the rate of soil respiration, but it also modifies the response of respiration to temperature, and there is mounting evidence that the temperature sensitivity of respiration decreases with higher temperatures and lower soil moisture levels^31–34^. Quantifying the interactions between various driving factors, especially temperature and humidity, on carbon fluxes is crucial for making more accurate predictions about the impact of climate change on carbon neutrality^15^.

Based on the aforementioned issues, we conducted a three-year continuous observation on NEE from 2018 to 2020 over the semi-arid grassland of the Loess Plateau in northwest China. During this period, we also estimated Reco and GPP. The main objectives of this paper were to evaluate: (1) the daily, seasonal, and interannual variations of carbon fluxes (GPP, Reco, and NEE), and (2) the combined effects of Ts, SWC, photosynthetically active radiation (PAR), VPD, precipitation, and normalized difference vegetation index (NDVI) on carbon exchange. We hypothesized that the semi-arid grassland is a moderate carbon sink within the ecosystem. And in addition to temperature and moisture, there are other factors that play a significant role in the changes in carbon fluxes, as previous studies have shown that the univariate linear regression results of temperature and moisture with carbon exchange were not satisfactory. A comprehensive understanding of the relationship between carbon exchange capacity and environmental factors in the region would complement the research on the carbon cycle of terrestrial ecosystems under global climate change, and provide an essential support and a theoretical basis for a thorough understanding of ecological reconstruction and restoration in practice, as well as for solving these problems.

## Materials and methods

### Site description

**Figure 1:**
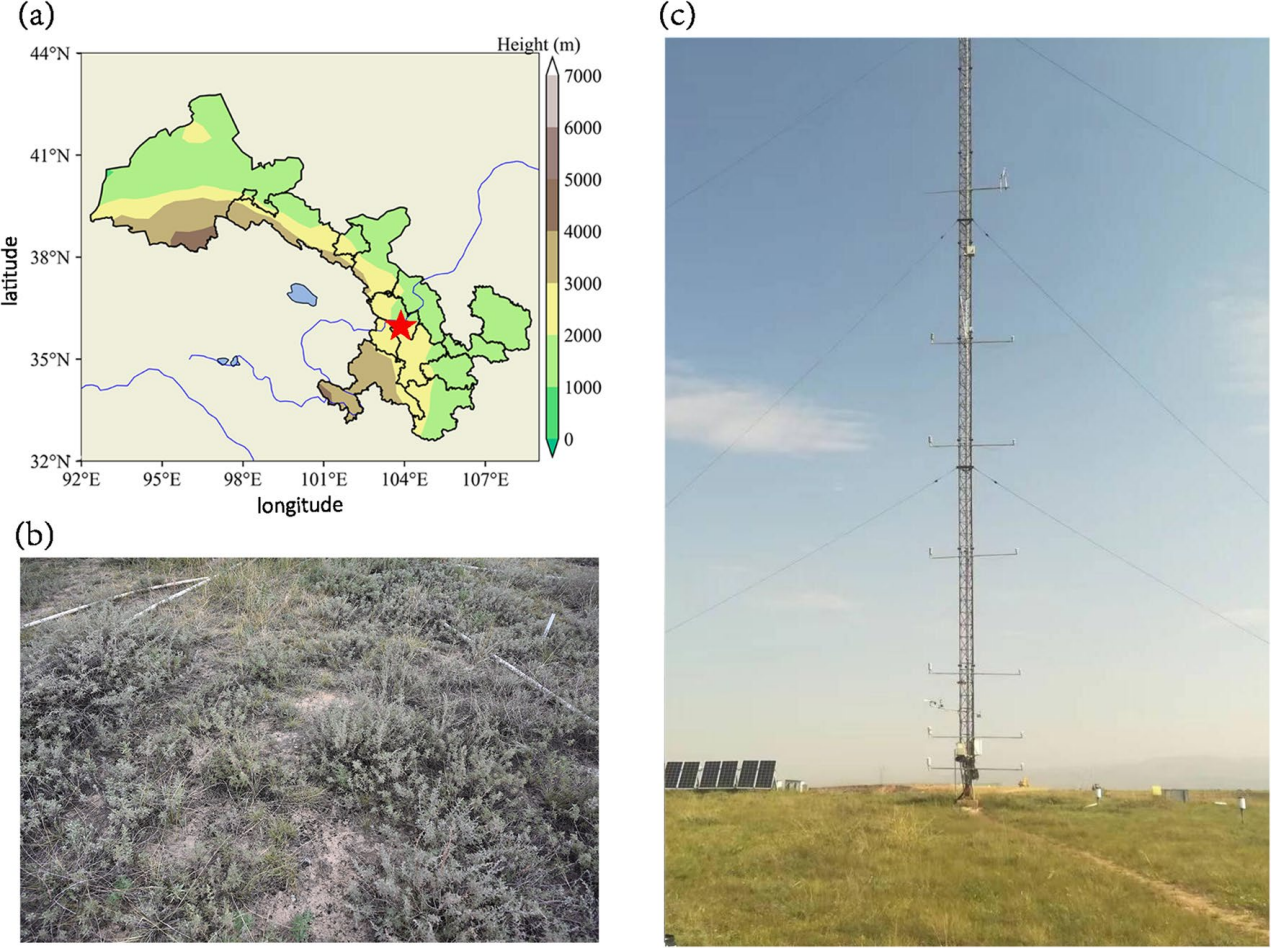
(**a**) Locations of the SACOL (Lat.: 35.946 ^o^N, Lon.:104.133 ^o^E, Alt.: 1970 m). This map is from Python 3 (https://www.python.org/). Panels (**b**) and (**c**) are photos of the underlying surface and the eddy covariance site at the SACOL during the growing season.

The experimental site is situated at the Semiarid Climate and Environment Observatory of Lanzhou University (SACOL), located on the top of Cuiying Mountain in Yuzhong Campus of Lanzhou University in Yuzhong County, Gansu Province (Lat.:35.946 °N, Lon.:104.133 °E, elevation 1965.8 m) (see Fig. 1). It belongs to semi-arid continental climate with annual mean air temperature of 6.7 °C and annual sunshine duration of about 2600 h. The average annual precipitation here is 382 mm, mainly from June to October. The annual evaporation is 1343 mm, with a frost free period of 90–140 days. The study area is characterized by typical residual hills, ridges, and gullies on the Loess Plateau, with a typical semi-arid grassland vegetation type. The average height of herbaceous vegetation in autumn is about 30 cm, followed by summer with an average height of 24 cm, the vegetation coverage reaches about 80% in summer and autumn, and the average plant height in spring is 15 cm. In winter, as the grassland vegetation evolves, the average plant height is only about 10 cm. The vegetation types are mainly Artemisia frigida, Stipa, and wild chrysanthemums, accompanied by some Camellia sinensis and dwarf wild cassia^35–37^.

### Instrumentation and measurements

The EC system was installed since 2017 to monitor the fluxes of CO_2_, water vapour, energy, and momentum. It consisted of a three-dimensional sonic anemometer (CSAT3, Campbell, USA) and an open-path CO_2_/H_2_O infrared gas analyzer (LI-7500, LI-COR, USA) mounted at 2.88 m above ground, the anemometer and the infrared gas analyzer supply digital output of the fluctuations in 3-D wind velocity, sonic temperature, water vapor and CO_2_ densit^38^. The data was collected with the frequency of 10 Hz, and the averaged values were calculated every 30 min, which would be stored in the data logger. The gas analyzers are calibrated by technical engineers using professional equipment in every spring to calibrate CO_2_, water vapor, and dew point values.

Along with the flux measurements obtained by the EC system, this study continuously measured standard meteorological and soil parameters with an array of sensors. Air temperature and relative humidity were measured with a temperature and relative humidity probe (HMP45C-L, Vaisala, Finland) mounted on the tower at the height of 2.0 m, then calculated the VPD according to those measurements. Soil temperature (STP01-L, Hukseflux, USA) and soil moisture (CS616-L, Campbell, USA) were measured at 5, 10, 20, 40, and 80 cm beneath the ground surface. A spectral reflectance sensor (SRS-NDVI, DECAGON, USA) was installed at 4 m above ground level for measuring NDVI. Precipitation was measured by a tipping bucket rain gauge at 0.3 m (52,202, Young, USA), each turn of the tipping bucket measured 0.1 mm precipitation. PAR was measured at 1.5 m above the ground, using a quantum sensor (LI-190R, LI-COR, USA). The instruments and measurement parameters used in this study are listed in Table 1 ^36,39,40^. All times reported in this article are in the Beijing time zone (Greenwich Mean Time + 8). The above instruments were installed in August 2017, the operation, calibration, and maintenance of the all instruments used followed the manufacturers’ standard procedures.

**Table 1:** The key instruments and measurements at SACOL during the whole period.

Instrument	Manufacturer, model	Measurements	Accuracy
Eddy covariance system	LI-COR, LI-7500RS	CO_2_ flux at 2.88 m	< 1%, 10 Hz
Soil temperature sensor	Hukseflux, STP01-L	Ts at 5, 10, 20, 40, and 80 cm depths, °C	± 0.02 ℃
Water content reflectometer	Campbell, CS616-L	SWC at 5, 10, 20, 40, and 80 cm, m^3^m^−3^	< 0.1% SWC
Tipping bucket rain gage	R.M Young, 52,202	Precipitation in mm	0.1 mm or ± 2%
Temperature and Relative Humidity Probe	Vaisala, HMP45C-L	Air temperature and humidity	± 0.10℃ and ± 1%RH
Quantum sensor	LI-COR, LI-190R	PAR in μmolm^−2^ s^−1^	± 1%, 400–700 nm
Spectral reflectance sensor	DECAGON, SRS-NDVI	NDVI at 650 nm and 810 nm	< 10%, field of view: 180°

### Data processing and quality control

Half-hourly NEE was calculated using EddyPro software (LI-COR, Lincoln, Nebraska, USA) based on the 10 Hz raw data (Details can be referred to https://www.licor.com/env/products/eddy_covariance/software.html). The following processing procedures were applied: (1) de-spiking: eliminate outliers that are far beyond reasonable values and have obvious errors due to instrument failures, weather effects, and random noise; (2) coordinate rotation: rotate the two-dimensional coordinate axis and calculate a series of statistics such as mean, pulsation, variance, and covariance, Then calculate the preliminary results of turbulent flux; (3) necessary corrections to the flux: such as density fluctuation correction (WPL)–the Webb, Pearman and Leuning density correction for effects of air density fluctuations on CO_2_ fluxes–to adjust air density changes caused by heat and water vapor fluctuations^41^, etc.; (4) quality control of flux data: including physical reasonable range testing, turbulence stationarity testing, and adequacy testing of turbulence development^42^, providing the flag “0” for high-quality fluxes, “1” for intermediate quality fluxes, and “2” for poor quality fluxes. Only fluxes flagged with “0” or “1” were adopted for further analysis. Based on the turbulence flux data obtained from the above processing, this study follows the standard correction method proposed by Papale et al. to perform data quality control on the NEE data of the site^43^. Positive value for NEE indicates that the region emits CO_2_ flux into the atmosphere, whereas negative value indicates that the region absorbs CO_2_ flux from the atmosphere.

Due to instrument failures and outlier data often resulting in missing observational data, it is necessary to fill in the missing data in order to examine the carbon balance of ecosystems. Two methods were used to fill in the missing data. Linear interpolation was used for gaps of 2 h or less, and the data gaps of less than 2d were filled using MDV method^44^ (mean diurnal variation), while the data gaps of greater than 2d were considered missing value and would not be filled in.

After filling in NEE data, we could calculate the corresponding Reco and GPP. We assumed that GPP is zero at nighttime, and the NEE between nighttime vegetation and atmosphere only comes from ecosystem respiration:

1 $$NEE_{night} = {\text{Re}} co_{night} .$$

Ecosystem respiration Reco can be defined as:

2 $${\text{Re}} co = {\text{Re}} co_{night} + {\text{Re}} co_{day} = NEE_{night} + {\text{Re}} co_{day} ,$$

wherein, Reco_night_ and Reco_day_ are ecosystem respiration at night and during the day respectively.

By fitting NEE nighttime data on a monthly basis and using the Reco function relationship established using nighttime Reco data, daytime Reco data can be obtained^33^.

Reco is mainly influenced by soil temperature and soil moisture^45^. This study uses (3) to fit the relationship between nighttime Reco and soil temperature and soil moisture, and uses 1-month time window^18^:

3 $${\text{Re}} co = a \times e^{{\left( {b \times T_{{\text{s}}} } \right)}} \times SWC^{c} .$$

Among them, SWC is the soil water content, Ts is the soil temperature, and a, b, and c are the parameters to be fitted.

Temperature sensitivity (Q_10_) is usually used to quantitatively describe the dependence of soil respiration process on soil temperature. The Reco was first parameterized with a traditional Q_10_ model to fit the soil temperature at 5 cm depth^44,46^:

4 $${\text{Re}} co = a \times e^{{b \times T_{{\text{s}}} }} ,$$

where a and b are regression coefficients.

The respiratory temperature sensitivity coefficient (Q_10_) is:

5 $$Q_{10} = e^{10 \times b} .$$

The GPP of an ecosystem can be defined as Ref.^47^:

6 $$GPP = {\text{Re}} co - NEE.$$

The VPD was calculated by the measurements of air temperature (Ta) and relative humidity (RH)^48^:

7 $$VPD = 0.611 \times e^{{\frac{17.27 \times Ta}{{Ta + 237.3}}}} \times \left( {1 - RH} \right).$$

## Results and discussion

### Meteorological conditions

**Figure 2:**
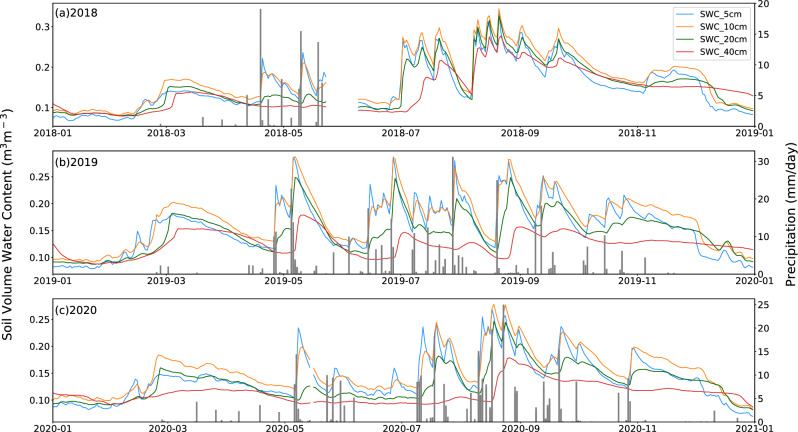
Time series of 30-min averaged soil volumetric water content (SWC in in m^3^m^−3^) at 5 cm, 10 cm, 20 cm, and 40 cm depths versus precipitation in mm/day at SACOL for (**a**) 2018, (**b**) 2019, and (**c**) 2020. Note that the precipitation data for June-December 2018 and from November 2019 to February are missing due to the instrument failure.

In the graph depicting changes in SWC at depths of 5, 10, 20, and 40 cm due to precipitation events in the region from 2018 to 2020, notable interannual and seasonal fluctuations in both precipitation and SWC were observed. It is worth noting that the precipitation data was unavailable for the periods from May 24th, 2018 to December 31st, 2018, and November 23rd, 2019 to February 17th, 2020 (Fig. 2). The precipitation is primarily concentrated from May to October, representing over 90% of the total precipitation for the year. The precipitation process exhibits a relatively consistent phase with solar radiation and surface temperature, meaning that rain and heat occur during the same period. This synchronization is highly beneficial for vegetation photosynthesis, growth, and metabolism, and consequently, it has an impact on carbon control. The seasonal variation of SWC is significant, with the highest SWC occurring in the middle of the growing season and the dormant season from November to April of the following year, during which there will be several snowfall events. The changes in SWC are generally consistent with precipitation. However, the upper layers of soil show a great sensitivity and responsiveness to precipitation events compared to the deeper layers. After rainfall triggers a soil moisture pulse, SWC in the upper layers decays at a faster rate than in the deeper layers. But, except for a day or two after rainfall, SWC at the depth of 5 cm is always less than 10 cm for the rest of time. This may be attributed to the rapid evaporation rate of water in the upper layers of the soil. Overall, precipitation events greater than 5 mm can impact the moisture content of the surface soil moisture at a depth of 10 cm, while precipitation events greater than 10 mm can affect the soil moisture at a depth of 20 cm. Precipitation events greater than 20 mm can affect soil moisture at a depth of 40 cm, and this impact has a lag of about 2 days. Different precipitation intensities will have a significant impact on the root respiration, decomposition, and metabolism processes of grassland vegetation in semi-arid areas. This, in turn, will adjust the diurnal and seasonal changes in carbon exchange. Previous studies have shown that precipitation less than 5 mm in arid and semiarid areas primarily affects SWC in the near-surface soil, and that precipitation events greater than 5 mm can effectively supplement moisture in the root layer moisture at greater depths in a desert soil zone^49^ These larger pulses are commonly referred to as "effective precipitation". Similar conclusion has been drawn on semi-arid grasslands.

It is worth noting that during the winter dormancy period, the SWC generally experiences a sudden increase in early March, reaching a maximum value of 0.15 to 0.20 m^3^·m^−3^, and then gradually decreases over time until the end of April. This is mainly attributed to the continuous melting of frozen soil as the temperature gradually increases, which in turn increases the soil moisture content of different layers.

**Figure 3:**
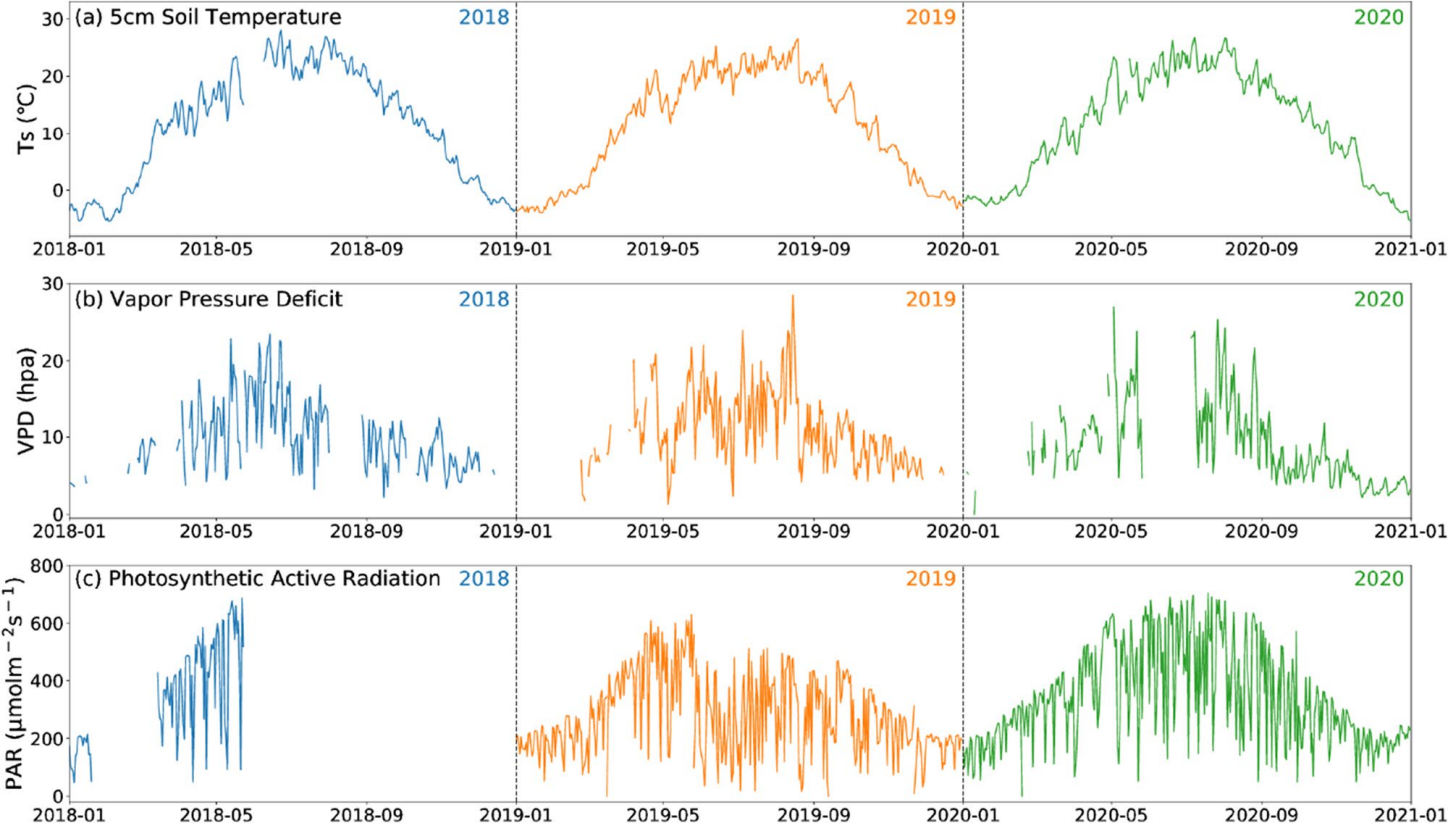
Temporal evolution of daily mean (**a**) soil temperature at 5 cm depth (Ts in °C), (**b**) vapor pressure deficit (VPD in hPa), and (**c**) photosynthetic active radiation (PAR in μmolm^−2^ s^−1^) at SACOL from January 2018 to December 2020. Different years are marked with different color lines. Light blue, yellow, and green lines show the years of 2018, 2019, and 2020, respectively.

Ts_5cm, VPD, and PAR displayed significant unimodal, intra-annual seasonality (Fig. 3). Interannual differences in meteorological variables were minimal, with an average daily Ts_5cm of 11.237 °C, and the average annual Ts_5cm were 11.09 ± 9.88, 11.22 ± 9.39, and 11.4 ± 9.24 °C in 2018, 2019, and 2020, respectively. The maximum values of Ts_5cm and SWC_5cm in 2018 were higher than in other observation years (the maximum values of daily average Ts_5cm and SWC_5cm in 2018 were 27.984 °C and 0.344 m^3^ $\cdot$ m^−3^, respectively; in 2019, they were 26.521 °C and 0.288 m^3^ $\cdot$ m^−3^; in 2020, they were 26.703 °C and 0.278 m^3^ $\cdot$ m^−3^). Throughout the observation period, the soil temperature stabilized above 15 °C after entering the growing season in May. This temperature range is conducive to plant growth. The maximum values of Ts_5cm and SWC_5cm occurred during the middle of the growing season, namely July and August. VPD is an important indicator that reflects the level of atmospheric drought, ranging from 1.81 to 28.49 hPa and follows a unimodal distribution, it is typically high during the growing season and low during the dormant season. VPD is related to the moisture content of the air and also varies with changes in precipitation^50^. Research has shown that the meteorological factors that dominate the interannual variation of VPD in semi-arid areas are temperature and absolute humidity^51^, which is reflected in the correlation coefficients between shallow soil temperature and VPD from 2018 to 2020 in this study, which were 0.642, 0.699, and 0.762 (p < 0.001), respectively. The maximum PAR were 685.66, 628.4 and 702.96 µmol/(m^2^·s) in 2018, 2019, and 2020, respectively. These levels were recorded during the growing period.

### Diurnal and seasonal variations of NEE, Reco, and GPP

**Figure 4:**
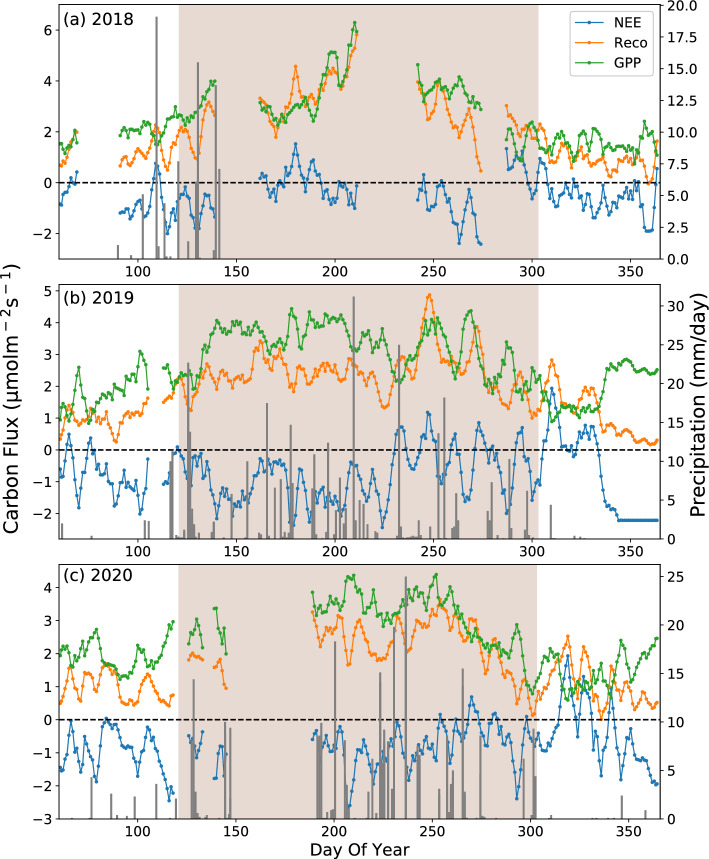
Daily mean of carbon fluxes in μmolm^−2^ s^−1^ (NEE, Reco, and GPP) versus precipitation in mm/day at SACOL for (**a**) 2018, (**b**) 2019, and (**c**) 2020. The solid curve shows the 5-day running averages of the carbon fluxes.

Strong seasonal variations in GPP, Reco, and NEE of the semiarid grassland on the Loess Plateau ecosystem were observed (Fig. 4, considering the growth of plants in the region during winter and the impact of low temperature on the quality of the instrument, this study did not use data from January and February of the observation years, and the shaded area corresponds to the growing period).

The CO_2_ release phenomena was most readily observed in winter and CO_2_ absorption was obvious in summer. This could be attributed to photosynthesis, which suggests that more plants are able to absorb more carbon during the growing season, in contrast to less photosynthesis occurring with fewer plants in the dormant season^7^. The period of high net CO_2_ uptake period occurred during the early growing season of 2019, which can be attributed to the higher amounts of rainfall that was experienced during the period. The NEE changes throughout 2019 and 2020 decreased in the early stage of the growing season, then began to rise in the middle of the growing season, and decreased again in winter, indicating an overall carbon sink state. The daily average NEE values are − 1.87, − 3.018 and − 2.93 g C/m^2^ for 2018, 2019, and 2020, respectively. The difference may be attributed to several factors, including the meteorological conditions of high temperature and humidity in the observation area in 2018, variations in the proportion and duration of missing data over the three-year observation period, or the influence of not taking into account the data from January and February.

In general, GPP and Reco exhibit single-peak seasonal changes every year, reaching their maximum values in the summer. Due to the rise in temperature and increased photosynthesis, which has been shown to enhance plant productivity^52^, the carbon exchange process in ecosystems dominated by carbon absorption has accelerated. In September (during the late growing season), both Reco and GPP decrease as the plants age and the temperature drops. Similar results have been found in arid desert grassland^53^, temperate desert grassland^54^, and temperate semi-arid sandy grassland in China^52^.

In the winter of 2019 and 2020, GPP showed a declining tendency at the beginning and then rose in late, while NEE showed the opposite trend (Fig. 4b,c). Maybe because at the end of the growing season in autumn, when plants begin to die and photosynthesis weakens, there is a decline in GPP and an increase in NEE. When deep winter arrived, the temperature continued to decrease, inhibiting respiration and causing GPP to exceed Reco. As a result, there was a decrease in NEE^55^. There was a study that has also shown that in winter, due to low air and soil temperatures, soil microorganisms, root respiration, and photosynthesis basically stop^56^. However, the solubility of CO_2_ in water increases as the decrease of temperature. At this time, CO_2_ in soil pores is readily absorbed by soil moisture and accumulates in the permafrost layer. CO_2_ concentration in the atmosphere diffuses towards the soil due to its higher concentration compared to that in soil pores, resulting in negative NEE values during winter. On the other hand, in the winter of 2020, we observed a slight increase in photosynthetically effective radiation (Fig. 2c), which may have also contributed to the increase in GPP during that period.

GPP increases after precipitation during the growing season. Precipitation promotes plant growth and provides better conditions for photosynthesis, leading to an increase in GPP. It means that the capacity of carbon sinks increases with higher levels of precipitation.

**Figure 5:**
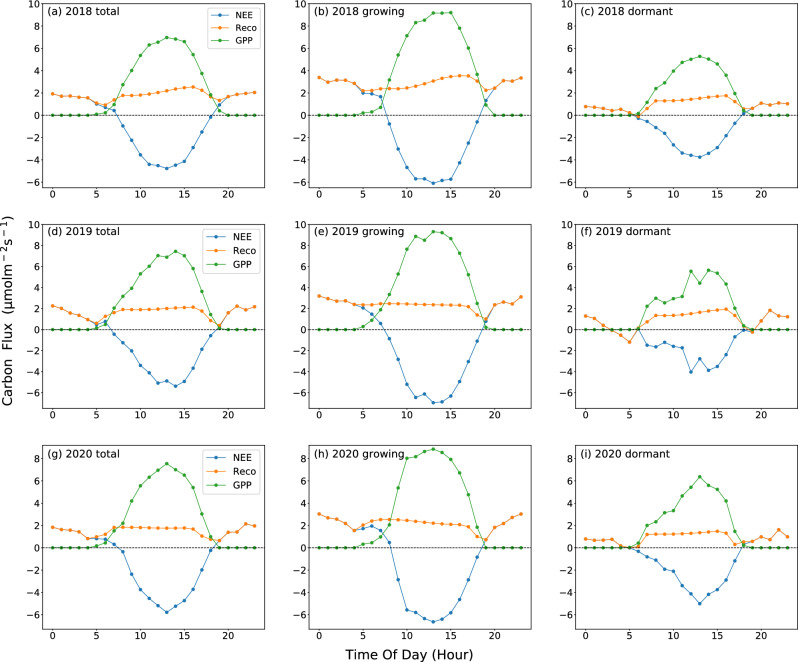
Diurnal variations of (**a**) one year-round average, (**b**) growing-season average, and (**c**) dormant-season average carbon fluxes in μmolm^−2^ s^−1^ (NEE, Reco, and GPP) at SACOL for 2018 (top pannel), 2019 (middle pannel, (**d**–**f**)), and 2020 (bottom panel, (**g**–**i**)), repectively.

The daily changes of GPP and NEE show an inverse "U" and "U" shaped variation curve (Fig. 5). During the daytime, as the light intensity increases, NEE gradually decreases (carbon absorption increases), reaching a negative value at 7:00 or 8:00, and peaking at noon (12:00 to 14:00). Then, it gradually increases until sunset when the ecosystem transitions from net carbon absorption to net carbon release (18:00 or 19:00). The variation of NEE is asymmetric, with a greater decrease in carbon absorption rate before reaching the peak than after reaching the peak. Because the photosynthesis process ceases to occur at night and respiration produces CO_2_ from the ecosystem, the ecosystem becomes a net source of CO_2_. With average overnight NEE values of 1.354, 1.182, and 1.181 µmol/(m^2^ $\cdot$ s) during the three years of observation, and average daytime NEE values of − 3.347, − 3.662, and − 3.7681 µmol/(m^2^ $\cdot$ s). Taking into account both daytime and nighttime carbon dioxide fluxes, the ecosystem was found to be a net carbon dioxide sink on a daily basis.

During the growing season, nighttime Reco is slightly higher than daytime Reco (the average daytime Reco values were 2.763, 2.22, and 2.183 µmol/(m^2^ $\cdot$ s) for 2018, 2019, and 2020, the corresponding nighttime Reco values were 3.048, 2.724, and 2.5 µmol/(m^2^ $\cdot$ s) respectively). There may be two reasons for this phenomenon: Firstly, soil respiration is dependent on photosynthesis, as the litter and root exudates released by plants are crucial for microbial metabolism. However, the carbon sequestered by photosynthesis is transported to the roots after a few hours and released through rhizosphere respiration at night^57,58^. Secondly, during the day, the air temperature is higher than the soil temperature, and the gas pressure is also higher. This can suppress the emission of soil CO_2_. But the soil temperature at night is higher than the air temperature, which facilitates the diffusion and release of soil CO_2_^59,60^.

The daily dynamics of NEE, GPP, and Reco in the dormant season are similar to those in the growing season, but the intensity of NEE and GPP in the dormant season is lower than that in the growing season. The peak values of NEE, GPP, and Reco during the observation period were − 4.27, 5.766, and 1.774 µmol/(m^2^ $\cdot$ s), respectively, which were significantly lower than the values during the growing season, which were − 6.955, 9.317, and 3.541 µmol/(m^2^ $\cdot$ s). The variation of Reco is a horizontal line approximately, but the respiration rate during the day is slightly higher than at night. Also observed from Fig. 5 is that overnight Reco was much higher during the growing season than during the dormant season, highlighting the importance of photosynthetic activity on the ecosystem respiration.

Some studies have shown that semi-arid grasslands have a significant carbon uptake function^17,18,61^. In this study, the average daily NEE were -0.492, -0.806 and -0.75 µmol/(m^2^ $\cdot$ s) in 2018, 2019 and 2020 (The significant difference in daily NEE between 2018 and other observed years may be due to excessive missing data during the dormant season). The average peak value during its observation period is -5.316 µmol/(m^2^ $\cdot$ s). The carbon absorption capacity of the semi-arid region of the Loess Plateau is higher than that of the Mongolian typical temperate continental short-grass steppe^62^ (the peak hourly value of NEE was -3.6 µmol/(m^2^ $\cdot$ s)) and the Horqin semiarid sandy land^60^ (the daily average of NEE is 0.14 ± 0.04 g C/(m^2^ $\cdot$ d)), but lower than the Mediterranean grazed grassland opening in a region of oak/grass woodland in California^33^ (the maximum value of NEE was − 4.8 g C/(m^2^ $\cdot$ d)), the typical steppe in Inner Mongolia^3^ (the mean of NEE was − 0.95 ± 0.31 µmol/(m^2^ $\cdot$ s)) and the semiarid desert steppe in northern China^63^ (the seasonal means of NEE was  − 3.09 µmol/(m^2^ $\cdot$ s)). Moreover, the carbon absorption capacity of grasslands in arid areas is significantly weaker than that in humid areas^64–67^. These differences may be due to differences in environmental factors such as radiation, temperature, and moisture, and on the other hand, plant species are also important factors affecting grassland ecosystem photosynthesis^52^.

### Effects of environmental factors on NEE, Reco, and GPP

**Figure 6:**
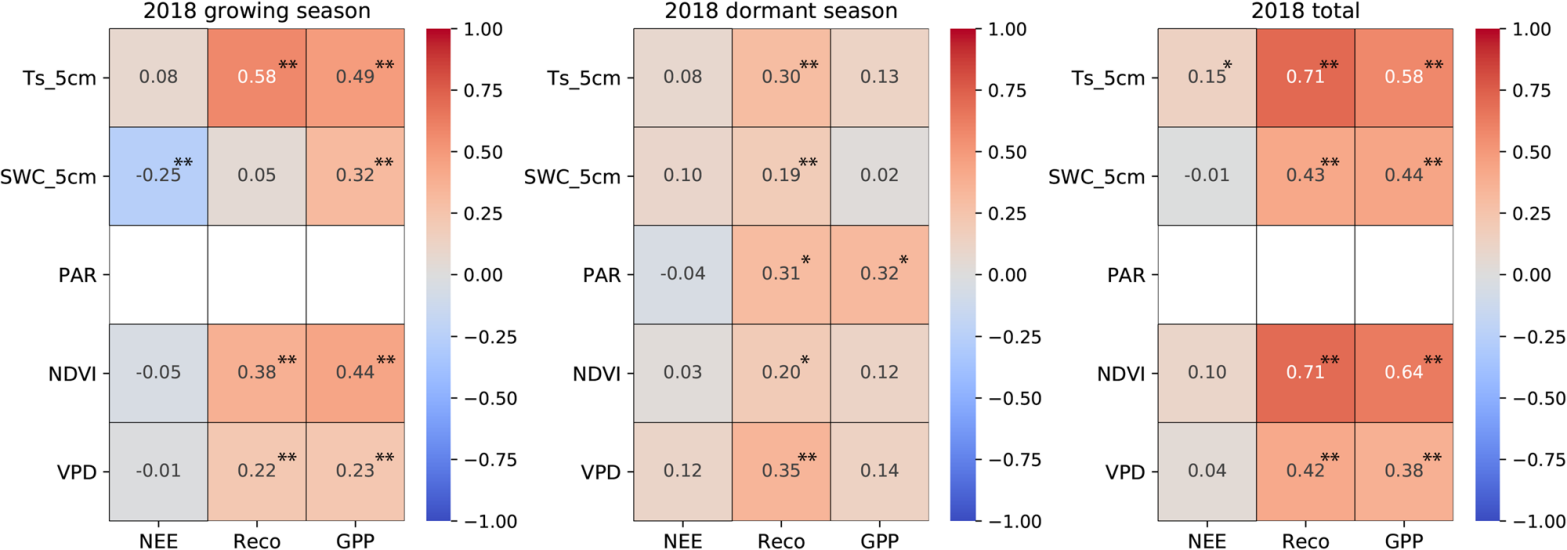
Pearson's correlation coefficients between the carbon fluxes (NEE, Reco, and GPP) and environmental factors (Ts_5cm, SWC_5cm, PAR, NDVI, VPD) for the growing season (left panel), dormant period (middle panel), and overall days (right panel) in 2018. The white square shows there is missing data. * denotes a significance level less than 0.05 (p < 0.05), and ** denotes a significance level less than 0.01 (p < 0.01).

**Figure 7:**
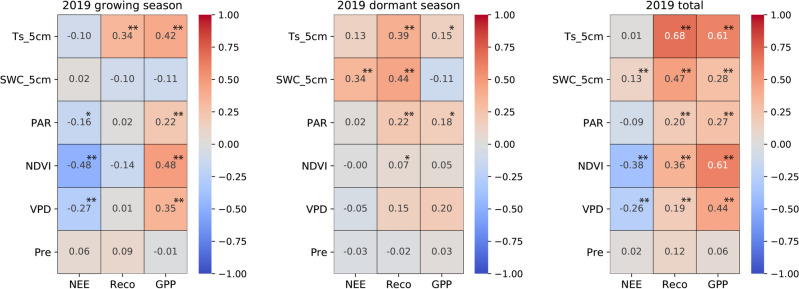
The same as Fig. 6, except for the year in 2019.

**Figure 8:**
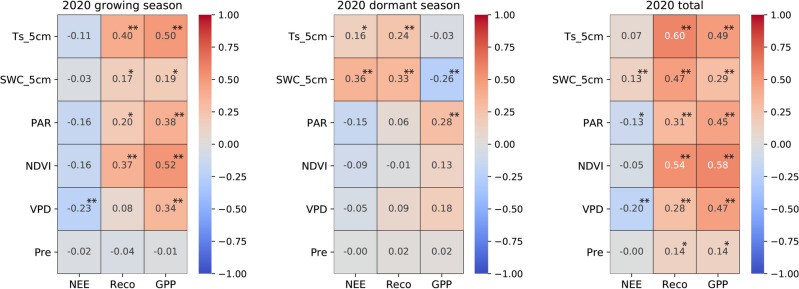
The same as Fig. 6, except for the year in 2020.

The carbon fluxes of the ecosystem are influenced by multiple environmental variables that primarily affect the biological and physical processes^67^. Based on the characteristics of single peak interannual variations similar to temperature in Reco and GPP, we have reason to speculate that soil temperature will have a significant impact on these two carbon fluxes (Figs. 3c and 4). The correlation analysis (Figs. 6, 7, 8) reveals the relationship between the daily average of environmental factors and carbon flux in different seasons. GPP and Reco were positively correlated with Ts_5cm for most of the time, Ts can explain 11.56–33.64% of the growing season Reco changes (p < 0.01) and 17.64% to 25% of the GPP changes (p < 0.01). In contrast, there was a weak negative correlation between NEE and Ts_5cm during the growing season of 2019 and 2020. This indicates that the response of GPP to Ts during the growing season was greater than that of Reco to Ts^60^ (Table 2). Similar results were also found in alpine meadows^55^. However, the impact of temperature on net carbon absorption is generally not significant, as indicated by low partial correlation coefficient and poor P-values, which is consistent with previous studies^17,22^. GPP and Reco are mostly positively correlated with SWC, while NEE in the growing season is negatively correlated with SWC. These results indicate that higher Ts and lower water stress during the growing season are beneficial for CO_2_ absorption, while higher soil temperature during the dormant season may reduce net CO_2_ absorption.

**Table 2:** Linear regression equations between carbon fluxes (Reco and GPP) and soil temperature at 5 cm depth (T_s_) at SACOL during the growing season from 2018 to 2020.

Year	Regression equation	R^2^	F
2018	Reco = − 0.099 + 0.157Ts	0.342**	66.669
	GPP = 1.032 + 0.117Ts	0.177**	27.634
2019	Reco = 1.079 + 0.069Ts	0.097**	17.793
	GPP = 1.517 + 0.093Ts	0.12**	24.59
2020	Reco = 0.616 + 0.087Ts	0.154**	25.355
	GPP = 0.862 + 0.118Ts	0.241**	44.054

**Indicates a significant difference at P < 0.01 level.

PAR, NDVI, and precipitation are negatively correlated with the daily average of NEE in most years and seasons. VPD and NEE showed a highly significant negative correlation (p < 0.01) during the growing seasons of 2019 and 2020, as well as throughout the entire year. In most years during the growing season, there is a strong positive correlation (p < 0.01) between PAR and NDVI with Reco and GPP. The high significance of PAR during the dormant season on GPP may prove that, as mentioned earlier, the abnormal increase in GPP in the winter of 2020 was indeed influenced by PAR. PAR and NDVI both indirectly affect NEE by influencing GPP and Reco.

VPD has a significant impact on the daily average Reco and GPP during the observation period, especially during the growing season (p < 0.01). From the calculation formula, it can be observed that VPD is more influenced by air temperature, so the relationship between VPD and temperature and carbon exchange is relatively consistent. Studies have shown a general upward trend in global VPD over the past 10 years^68^, and VPD in the semi-arid region of northwest China is relatively high compared to other regions^51^. We speculate that in the future, as drylands continue to expand^69^, VPD may have a significant impact on global carbon sequestration.

**Figure 9:**
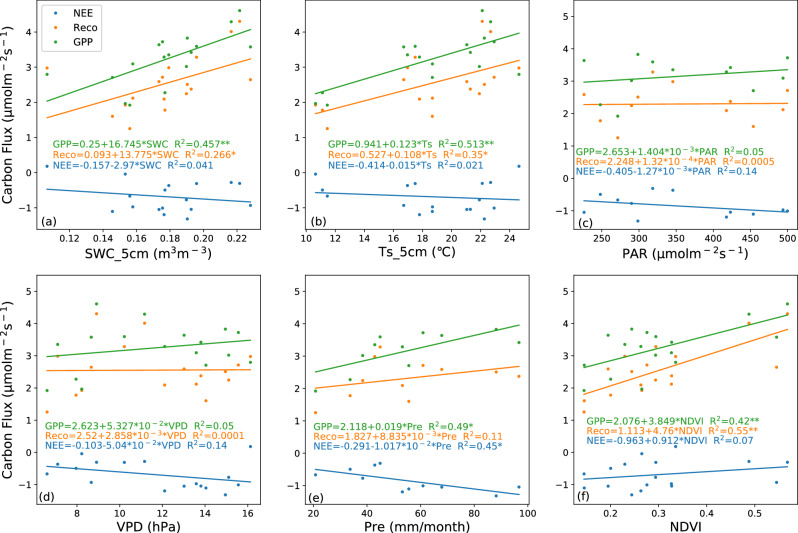
Relationship between monthly carbon fluxes (NEE, Reco, and GPP) and environmental factors at SACOL during the growing seasons from 2018 to 2020, (**a**) SWC_5cm, (**b**) Ts_5cm, (**c**) PAR, (**d**) VPD, (**e**) precipitation, and (**f**) NDVI. The light blue, yellow, and green solid lines represent the optimal linear fitted lines for NEE, Reco, and GPP, respectively.

Carbon fluxes are affected by numerous complex and interconnected environmental factors, which exhibit significant variations across different time scales^12,70^. The increase in precipitation enhances soil moisture in the semi-arid area, stimulates plant growth and microbial activity, and promotes the transfer of carbon from aboveground plant components to the roots. During the growing season, GPP and Reco were positively correlated with Pre, while NEE was negatively correlated with Pre (Fig. 9e). The response of soil moisture to precipitation during the growing season is sensitive (Fig. 2). NEE and SWC_5cm showed a negative correlation, but GPP and Reco were positively correlated with SWC_5cm. Additionally, GPP was found to be more sensitive to soil water availability compared to Reco. It showed different slopes when plotting the linear fitting diagram (Fig. 9a), which is consistent with the research results of semi-arid temperate grasslands^71^ and desert grasslands^72^. That is because the main impact factors on NEE and GPP are plants, changes in Reco are mainly influenced by the carbon availability of the soil carbon pool and microbial activity^73^. On semi-arid grasslands, plant roots are typically found in the topsoil layer, therefore the deep soil water supply of grasses is limited, and GPP is more limited by drought than Reco^74^. As for soil temperature, there is a significant positive correlation between GPP and Reco and soil temperature during the growing season (Fig. 9b). The correlation between the monthly average value of PAR and carbon fluxes (NEE, Reco, and GPP) are not significant (Fig. 9c).

VPD is negatively correlated with NEE, possibly due to the sensitivity of stomata to VPD^75^, which leads to stomatal closure under drought conditions. The coefficient of determination between VPD and NEE is greater than that of temperature, indicating that VPD is more influential than Ts in driving NEE under drought conditions. Both GPP and Reco show a positive correlation with VPD, but neither variable is statistically significant. this indicates that VPD is not the primary factor influencing the monthly values of Reco and GPP during the growing season (Fig. 9d). This is contrary to the results in section "Effects of environmental factors on NEE, Reco, and GPP", this indicates that the short-term impact of VPD on carbon exchange abilities during the growing season is more important than the long-term impact.

NDVI is positively correlated with GPP and Reco (Fig. 9e,f), and GPP has a stronger response to precipitation than Reco, indicating that changes in water availability have a greater impact on plants than on soil microorganisms. Therefore, the increase in GPP may be attributed to the increase in vegetation caused by precipitation (R = 0.439). During the growing season, the carbon sink capacity in the semi-arid area of the Loess Plateau increases with higher precipitation and decreases with lower precipitation. This study found that NDVI has a more significant impact on the monthly average of Reco and GPP compared to the daily average (Figs. 6, 7, 8). This is evident from the coefficients of determination and P-values. This indicates that NDVI affects the long-term changes in carbon exchange capacity between these two carbon fluxes.

**Figure 10:**
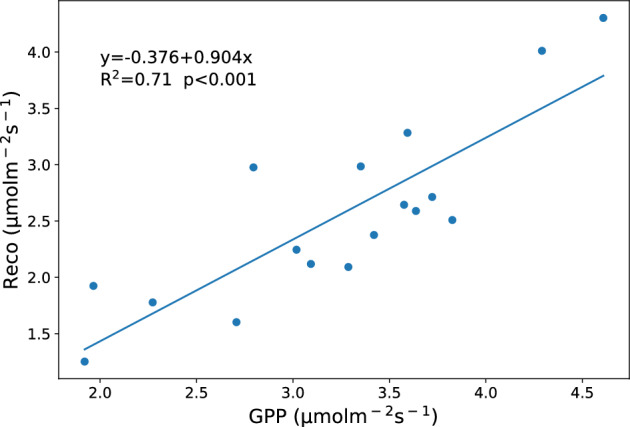
Relationship between monthly value of Reco and GPP at SACOL during the growing seasons from 2018 to 2020. The blue solid line show the linear fitted line.

In addition to SWC, Ts, precipitation, and biomass, Reco is also a significant factor in explaining GPP during the growing season^76,77^. The result of the linear regression model (Fig. 10) indicates that the contribution of GPP to the variation of Reco is 70.6%. Reco increases with the increase of GPP. Photosynthesis and respiration are tightly coupled processes, plant respiration relies on stored carbohydrates, while primary production supplies the carbon substrate for respiration. That is to say, photosynthetic products regulate the carbon cycle above and below the ground in semi-arid grasslands. Similar results have been observed in the semiarid desert steppe and shrubland of China^6,15,63,72^, the Mediterranean C3/C4 grassland^33,78^ and oak–grass savannah^79^.

### The interaction between soil temperature and moisture and ecosystem carbon fluxes

During the three years of observation, a highly significant positive linear relationship was found between Ts and SWC (2018: R = 0.53, p < 0.001; 2019: R = 0.551, p < 0.001; 2020: R = 0.561, p < 0.001). Low SWC occurs below 0 ℃, while medium SWC occurs between 0 and 15–18 °C. High moisture conditions typically coincide with high temperature, while low temperatures and drought often occur simultaneously. Some studies predict that the interaction between moisture and temperature has a stronger impact on ecosystem carbon flux^80,81^.

**Figure 11:**
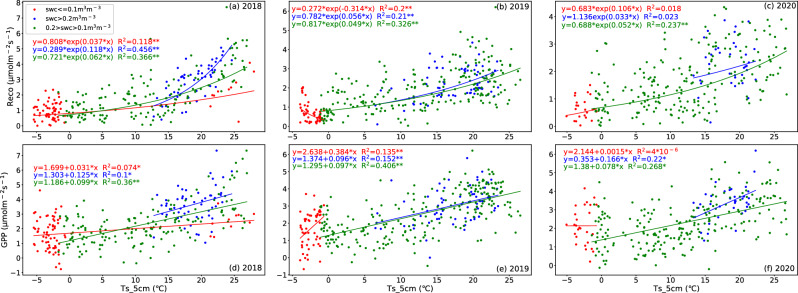
The scattering plots of Reco versus Ts_5cm (top panel) and GPP versus Ts_5cm (bottom panel) under different soil moisture conditions at SACOL for 2018 (left), 2019 (middle), and 2020 (right), respectively. The red solid circles denote the dry soil conditions (SWC_5cm ≤ 0.10m^3^m^−3^). The blue solid circles denote the wet soil conditions (SWC_5cm ≥ 0.20m^3^m^−3^). The green solid circles denote the moderate wet soil conditions (0.10 m^3^m^-3^ < SWC_5cm < 0.20m^3^m^−3^). The solid curves indicate the corresponding fitted lines.

In most cases, Reco shows exponential growth with the increase of Ts (P < 0.01). When SWC < 0.1 m^3^ m^−3^, Reco slowly increases with the increase of Ts (average Q_10_ = 1.459 during the observation year). When SWC ≥ 0.2 m^3^ m^−3^, Reco rapidly increases (Q_10_ = 2.132). This indicates that a low SWC can inhibit the sensitivity of Reco to Ts. Moreover, the correlation coefficient between GPP and Reco and Ts is higher when SWC is high (Fig. 11). Some studies show that drought conditions can lead to a decoupling of soil respiration and temperature^29^. That is to say, under drought conditions, carbon exchange is still very low even under suitable temperature conditions. As long as moisture conditions are favorable, GPP will rapidly increase with the increase in Ts. However, in this study, the impact of drought stress on GPP was not significant, but it did have an effect on Reco. This may be because the dry litter layer and upper soil are the sites for most heterotrophic respiration, whereas photosynthesis can draw water support from the roots in deeper soil^82^.

**Figure 12:**
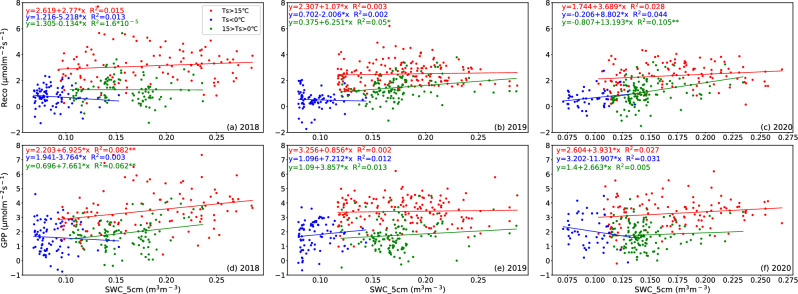
The same as Fig. 11, except for the scattering plots of Reco versus SWC_5cm (top panel) and GPP versus SWC_5cm (bottom panel) under different soil moisture conditions at SACOL for 2018 (left), 2019 (middle), and 2020 (right), respectively.

In most cases, when the temperature is above zero, GPP and Reco are more sensitive to temperature than when it is below zero (Fig. 12). At the temperature above zero, the response of Reco and GPP to SWC in 2018 is minimally affected by different temperature conditions. Similarly, the response of GPP to SWC in 2019 and 2020 is also unaffected by temperature conditions. High temperatures suppressed the response of Reco to SWC in 2019 and 2020, but no such phenomenon was observed in 2018. This difference may be related to the varying soil moisture conditions observed from 2018 to 2020. Therefore, appropriate SWC can alleviate low-temperature stress and maintain high GPP and Reco values. However, even under appropriate temperature conditions, as long as the SWC is low, GPP and Reco will be significantly inhibited. Therefore, from the perspective of ecosystem productivity, the potential harm of water stress on the GPP is greater than that of low-temperature stress^83^, which is consistent with the findings in alpine meadow^50^.

Water and temperature are often considered as two major abiotic factors that influence the ecosystem carbon exchange processes in grassland ecosystems^21,33^. During the growing season, the multiple linear regression results of carbon exchange and various meteorological factors (Ts_5cm, SWC_5cm, PAR, and VPD) from 2018 to 2020, considering the interaction between temperature and moisture (Ts_5cm * SWC_5cm), showed a significant increase in the coefficient of determination of the models in these tables (Tables 3, 4 and 5) compared to the models that did not consider the interaction between temperature and humidity (not provided in the text). The increase in the coefficient of determination was particularly notable for Reco and GPP, and the majority of these models passed the significance test (p < 0.01). From these tables, it can be seen that the combination of multiple meteorological factors simultaneously explains the highest proportion of changes in Reco (up to 46.1%) and GPP (up to 33.87%). So, it is necessary to consider the interaction between SWC and Ts when establishing carbon exchange equations in the study region.

**Table 3:** Multiple regression equations and curve fitting analyses between carbon fluxes (NEE, Reco and GPP) and environmental factors (Ts, SWC, PAR, VPD) at SACOL during the growing season in 2018.

Carbon flux	Regression equation	R^2^	F
NEE	NEE = 6.78 − 0.281Ts − 47.689SWC + 2.001Ts*SWC	0.126**	6.05
	NEE = 6.839 − 47.274SWC − 0.241Ts − 5.2*10^−2^VPD + 1.908Ts*SWC	0.138*	4.991
Reco	Reco = 6.998 + 2.366Ts*SWC − 45.531SWC − 0.207Ts	0.457**	6.898
	Reco = 7.024 − 45.35SWC − 0.189Ts + 2.27*10^−2^VPD + 2.326SWC*Ts	0.461**	26.664
GPP	GPP = 0.218 + 0.074Ts + 2.158SWC + 0.366Ts*SWC	0.308**	18.67
	GPP = 0.185 + 0.052Ts + 1.925SWC + 2.93*10^−2^VPD + 0.418Ts*SWC	0.312**	14.187

*Indicates a significant difference at P < 0.05 level.

**Indicates significant difference at P < 0.01.

**Table 4:** The same as Table 3, except for the growing season in 2019.

Carbon flux	Regression equation	R^2^	F
NEE	NEE = − 2.085 + 0.218Ts − 9.265VWC − 0.1VPD − 0.837Ts*VWC	0.11**	5.527
	NEE = 0.018 − 0.002PAR − 0.1Ts*VWC	0.05*	4.542
	NEE = − 1.164 − 0.598Ts*VWC + 4.902VWC + 0.164Ts − 0.001PAR − 0.1VPD	0.115**	4.568
Reco	Reco = 3.037 + 0.508Ts*VWC − 10.298VWC − 0.025Ts	0.104**	6.898
	Reco = 2.686 − 8.397VWC + 0.067Ts + 0.269VWC*Ts − 0.1VPD	0.172**	9.232
	Reco = 3.936 + 0.705Ts*VWC − 0.061Ts − 14.464VWC − 4*10^−4^PAR	0.124**	6.241
	Reco = 2.836 + 0.065Ts − 10.78VWC + 1.38*10^−3^PAR − 0.107VPD + 0.345Ts*VWC	0.208**	9.244
GPP	GPP = 3.858 + 0.796Ts*VWC − 0.062Ts − 13.929VWC + 1.6*10^−3^PAR	0.2**	10.938
	GPP = 4.469 + 0.902Ts*VWC − 16.032VWC − 0.071Ts	0.135**	9.28
	GPP = 4.77 − 17.662VWC − 0.151Ts + 1.107Ts*VWC + 6.67*10^−2^VPD	0.169**	9.037
	GPP = 3.999 − 15.682VWC − 0.099Ts + 2.5*10^−3^PAR + 5.41*10^−3^VPD + 0.943Ts*VWC	0.225**	10.194

**Table 5:** The same as Table 3, except for the growing season in 2020.

Carbon flux	Regression equation	R^2^	F
NEE	NEE = − 3.622 − 1.26Ts*VWC + 20.218VWC + 0.237Ts − 8.2*10 − 4PAR − 6.72*10^−2^VPD	0.087*	2.538
	NEE = 0.891 + 3.72*10 − 4PAR + 0.376Ts*VWC	0.154**	12.622
Reco	Reco = − 2.655 − 0.797Ts*VWC + 20.464VWC − 0.21Ts	0.21**	12.15
	Reco = − 3.252 − 0.996VWC*Ts + 21.953VWC + 0.303Ts − 6.29*10^−2^VPD	0.242**	10.841
	Reco = − 2.65 − 0.795Ts*VWC + 0.211Ts + 20.441VWC − 5.1*10^−5^PAR	0.21**	9.049
	Reco = − 3.31 + 0.313Ts + 20.789VWC + 1.94*10^−3^PAR − 0.118VPD − 1.024Ts*VWC	0.328**	13.004
GPP	GPP = 0.185 + 0.296Ts*VWC + 0.076Ts + 2.9449VWC	0.248**	15.07
	GPP = 0.185 + 0.296Ts*VWC + 2.893VWC + 0.077Ts − 1.79*10^−3^VPD	0.248**	11.221
	GPP = 0.522 + 0.846VWC + 0.049Ts + 0.298Ts*VWC + 1.34*10^−3^PAR	0.32**	16.048
	GPP = 0.312 + 0.571VWC + 0.075Ts + 2.76*10^−3^PAR + 5.1*10^−2^VPD + 0.236Ts*VWC	0.307**	13.619

## Conclusions

Based on the eddy covariance technique, this study quantified the three-year-long carbon exchange fluxes over the Loess Plateau semi-arid grassland of northwest China. The study also analyzed the variations in these fluxes and identified the driving factors. The main conclusions can be summarized as follows:

1. The daily average NEE values were − 1.87, − 3.018, and − 2.93 g C/m^2^ for 2018, 2019, and 2020, respectively. The annual cumulative average NEE value was − 0.778 kg C/m^2^, and the cumulative value during the growing season accounted for approximately 83.81%. Overall, the semi-arid grassland was proven to be a moderate carbon sink within the ecosystem.
2. Ts_5cm and NDVI were identified as two significant environmental influencing the daily variation of Reco (R_Ts_5cm_ = 0.71, R_NDVI_ = 0.71) and GPP (R_Ts_5cm_ = 0.71, R_NDVI_ = 0.61) during most integration periods (p < 0.01). Additionally, NDVI had a strong indirect impact on NEE due to its high correlation with GPP. As for the monthly values, Ts_5cm and SWC_5cm showed significant positive correlations with Reco (R_Ts_5cm_ = 0.59, R_SWC_5cm_ = 0.52) and GPP (R_Ts_5cm_ = 0.72, R_SWC_5cm_ = 0.68) during the growing season (p < 0.01). However, apart from precipitation, the correlation between NEE and other driving factors was very weak.
3. Reco increased with GPP (R = 0.84, p < 0.001), indicating a strong coupling between photosynthetic C uptake and respiratory C loss, and photosynthetic products regulate the carbon cycle both above and below the ground in semi-arid grasslands.
4. Compared to dry soil conditions, the temperature sensitivity of Reco (Q_10_) under wet soil conditions increased by 46.13%, and the potential impact of water stress on the GPP was greater than that of low temperature stress. In addition, considering the interaction between soil temperature and soil moisture would help in constructing the carbon exchange calculation model.

In the context of future dryland expansion, these results provide a valuable experience for future work in predicting and estimating carbon sources and sinks in typical semi-arid regions. To further confirm the impact of various environmental factors on different carbon fluxes, future research can integrate eddy covariance techniques, soil respiration measurements, and isotope analysis. This will allow for a more comprehensive analysis and examination of the relevant mechanisms and processes.

## Data Availability

The data that support the findings of this study will be available from the corresponding author, J. Bi, following a 6-month embargo from the date of publication.
